# A Case Series of Late Vascular Lesions of Traumatic Etiology: Endovascular and Surgical Approaches

**DOI:** 10.7759/cureus.39457

**Published:** 2023-05-24

**Authors:** Gustavo Melo-Guzmán, Erik Burgos-Sosa, Andres Camilo Granados-Hernández, Rossy Taveras, Laura Sanchez-García, Fernando Espinosa-Lira

**Affiliations:** 1 Neurology Endovascular Therapy, Hospital Juárez de México, Mexico City, MEX

**Keywords:** ns: neurosurgery, endovascular procedures, dural fistula, pseudoaneurysm, arteriovenous fistula, traumatic vascular injurie

## Abstract

There is a broad spectrum of pathology in traumatic vascular injury. Arteriovenous fistula (AVF) is an abnormal communication between the high-flow arterial system and the low-flow venous network, directly connecting the afferent artery and nearby draining veins without the regular intervention of the capillary bed. Most of these fistulas occur due to incidental or iatrogenic injury. A retrospective review of procedures performed by an endovascular surgeon in a tertiary center identified 15 cases of vascular injuries that encompassed all these different clinical scenarios, including post-traumatic, iatrogenic, or spontaneous origin. The information collected, including patient age, sex, previous symptoms, and treatment, was gathered from medical records. In addition, information on procedural technique, endovascular devices used, and specific intraprocedural details were collected from procedure notes and angiographic images. A broad spectrum of injuries can present as late trauma complications (over three months); endovascular treatment is a safe and effective approach for intracranial and extracranial injuries. Endovascular treatment can be a sole option or adjuvant to other hybrid therapies and has emerged as essential for treating these lesions as a first option. We have described standard techniques to treat different vascular pathologies, sometimes with limited resources.

## Introduction

There is a broad spectrum of pathology in traumatic vascular injury, which may range from simple intimal tearing to complete rupture of any vessel; from this scenario, several complications may present, including dissection between the three layers of the vessel producing active arterial extravasation in this false lumen resulting in a pseudoaneurysm or the abnormal communication between the arterial and venous systems. Arteriovenous fistula (AVF) is an abnormal communication between the high-flow arterial system and the low-flow venous network, which directly connects the afferent artery and nearby drainage veins without the regular intervention of the capillary bed [1]. Most fistulas occur because of incidental or iatrogenic injury [2-4].

Cerebral pseudoaneurysms are rare lesions comprising 1% of intracranial atherosclerosis, their mortality profile is significant, and their management is challenging [5,6]. Traumatic pseudoaneurysms are more common. They usually present with acute or delayed epidural hematoma and are often associated with additional subdural, subarachnoid, or intracerebral hemorrhage. Aneurysm rupture sometimes occurs up to several years after injury [5]. Pseudoaneurysms often become symptomatic, producing rapid and potentially fatal hemorrhage [7]. Given their unpredictable bleeding risk, pseudoaneurysms should be treated promptly in most cases, and most can be repaired with minimally invasive endovascular techniques that are safe and effective [5]. To treat symptomatic dissections of extracranial vessels associated with pseudoaneurysms, stents and coils have been used to preserve the parent artery [8]. The advent of flow-diversifying stents such as the Pipeline™ (Medtronic PLC, Dublin, Ireland) makes it possible to preserve the main vessel of a pseudoaneurysm while promoting intraluminal remodeling and vessel reconstruction [9].

## Materials and methods

Fifteen cases of vascular lesions from different clinical scenarios, including posttraumatic, iatrogenic, or spontaneous origin, were identified through a retrospective review of procedures performed by one endovascular surgeon from the Department of Endovascular Neurosurgery of the Hospital Juarez de México, Mexico City, Mexico.

Information, including patient age, sex, past symptoms, and treatment, was collected from the medical records. Information regarding procedural technique, endovascular devices, and specific intraprocedural details were gathered from procedural notes and angiographic imaging. Patient de-identification was done for the database. The Research and Teaching Directorate at Hospital Juarez de Mexico, Mexico, approved all data collection and analysis methods (approval number: 035/22).

In most cases, the endovascular approach warranted definitive treatment. However, some patients required surgical or even hybrid treatment. This report is organized, presenting the extracranial pseudoaneurysms and arteriovenous fistulas and, in the second part, the intracranial dural fistulas and pial fistulas.

## Results

Pseudoaneurysms

Extracranial pseudoaneurysms and fistulas originating from the carotid artery and its branches are uncommon lesions with post-traumatic, iatrogenic, or spontaneous origin [3,4].

Case 1. Left Subclavian Artery Pseudoaneurysm 

**Figure 1:**
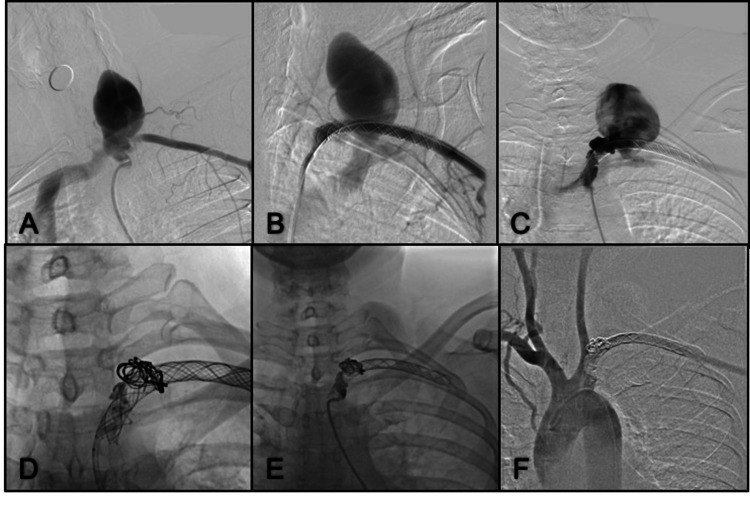
Oblique left thorax projection angiogram from the left subclavian artery (A) A large pseudoaneurysm and early opacification of the innominate vein are demonstrated; (B) The sac was still filling after the covered stent placement in the left subclavian artery; (C) There was still leakage after placing a second stent, covering the proximal third of the subclavian artery; (D) A microcatheter was navigated through the leakage site, where large coils were deployed at the neck of the pseudoaneurysm. Then Onyx® was slowly administered so as not to go further into the coil mesh; (E) Final Onyx cast is shown between the coil mesh and surrounding the proximal stent; (F) Final control with no aneurysm filling or leakage.

A 58-year-old female patient with a clinical history of diabetes mellitus type II and breast adenocarcinoma, presented with increased volume in the left neck after the installation of a port-a-cath device for chemotherapy. Computed tomography angiography (CTA) showed the presence of a left subclavian artery pseudoaneurysm associated with an arteriovenous fistula draining to the left subclavian vein. A covered stent (Wallgraft 8 mm x 50 mm) was successfully deployed in the left subclavian artery. One week later, a control angiogram showed a proximal stent endoleak, a new stent (9 mm x 70) mm was overlapped, and there still was an endoleak. Using a microcatheter (Apollo™ and Marathon™, Medtronic PLC), we deployed coils across the leak and then Onyx® (using ethylene vinyl alcohol copolymer (EVOH)) using the coils as a mesh so that there was no migration of the embolic agent into the vein (Figure 1). Further, angiographic checks revealed no filling of the sac and no leaks. The patient received double antiplatelet therapy consisting of 300 mg of aspirin and 600 mg of clopidogrel.

Case 2. Brachiocephalic Trunk Pseudoaneurysm

**Figure 2:**
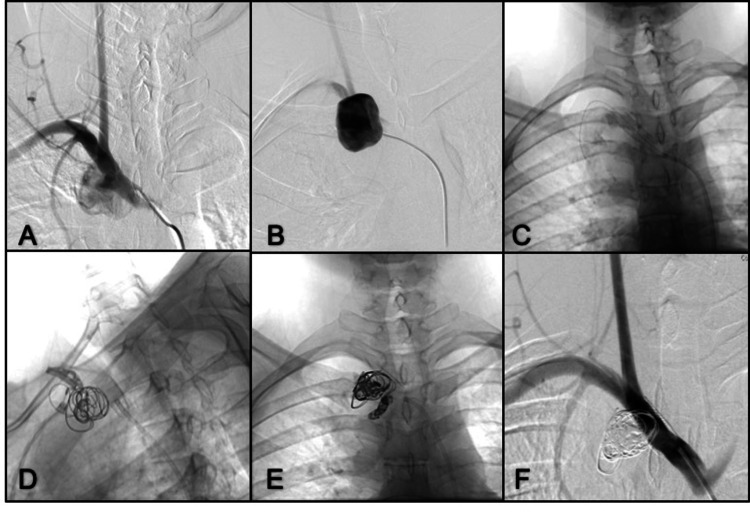
DSA AP projection in the early arterial phase (A) A giant pseudo aneurysm of the brachiocephalic trunk is seen; (B) Thoracic AP projection in the late arterial phase showing the complete filling of the pseudo aneurysm sac; (C) WALLSTENT™ (Boston Scientific Corporation, Marlborough, Massachusetts, United States) deployed on the brachiocephalic trunk; (D) Oblique view shows a microcatheter was advanced into the struts, and coils were deployed in the neck of the pseudoaneurysm; (E) Onyx was administered into the coil mesh only near the neck with a controlled slow injection; (F) Complete occlusion of the pseudoaneurysm and reconstructed brachiocephalic trunk. DSA: digital subtraction angiography; AP: anterior-posterior

A 38-year-old man presented with blurred vision, phosphene perception, intense headache, left arm acrocyanosis and paresthesia, syncope, and right commissure deviation. Initial tomography identified an ischemic stroke in the left parietal lobe. CTA and angiogram identified a giant pseudoaneurysm in the proximal edge of the brachiocephalic trunk (Figure 2). The sac measured was 66 mm x 86 mm with a 12 mm neck. We deploy a stent (WALLSTENT™ 9mm x 30 mm, Boston Scientific Corporation, Marlborough, Massachusetts, United States) into the brachiocephalic trunk. Then navigated a microcatheter (MACH 1™, Boston Scientific Corporation) inside the pseudoaneurysm and deployed three helical coils, and, finally, one vial of liquid embolic (Onyx) using a similar technique mentioned in Case 1 with a slow injection of liquid embolic to use the coil mesh to prevent migration into the rest of the sac and occluding only the neck. The patient received double antiplatelet therapy consisting of 300 mg of aspirin and 600 mg of clopidogrel. Further controls revealed no residual or recurrent lesions.

*Case 3. Internal Cervical Carotid Artery Giant Saccular Aneurysm * 

**Figure 3:**
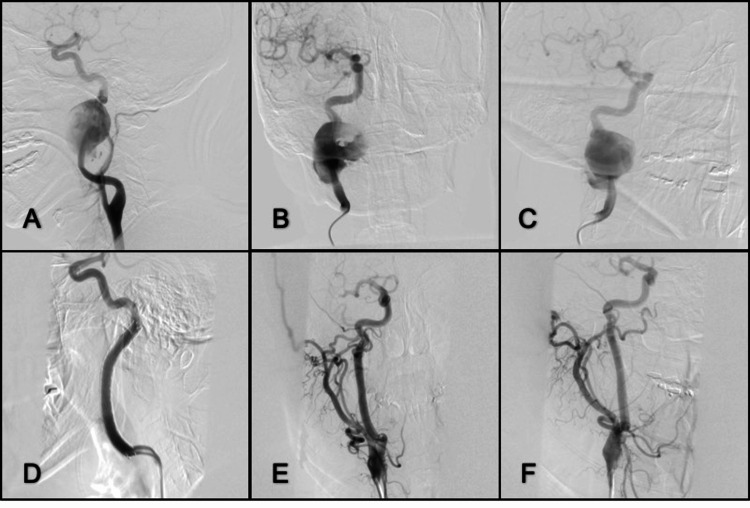
Lateral, anterior-posterior, and oblique right projection from the right common carotid artery (A) Lateral, (B) anterior-posterior, and (C) oblique view of the right common carotid artery before treatment; (B) Giant saccular aneurysm at the middle third of a C1 segment; (D) Lateral projection of right internal carotid after stent deployment; (E, F) Anterior-posterior and oblique projection of the common carotid artery show complete aneurysm occlusion at the two-year follow-up.

A 42-year-old man with no history of traumatic lesions developed a pulsatile lesion in the right pharynx region. This mass had appeared gradually over three years, accompanied by cough and dysphonia. He did not have any neurological impairment. An angiography was performed, which reported a giant saccular aneurysm of the right internal carotid in its C1 segment (Figure 3). An angioplasty was performed with sole stenting reaching 70% of an aneurysm thrombosis with no impairment of the pattern vessel. Three months later, another angiography was performed, which reported a giant saccular aneurysm in the medium third of the right internal carotid (33 mm x 30 mm). Fifteen months later, a third angiography was performed, which reported thrombosis of 90% of aneurism. Then, a year later, a fourth angiography was performed, which reported thrombosis of 80% of the aneurysm and leaks in the proximal and distal thirds of the rebuilt vessel. Finally, an angioplasty was served with a self-expanding stent. Two years later, another angioplasty was required because of a stent fracture. The angiography reported thrombosis of 100% of the aneurysm. The patient remained symptom-free eight years postoperatively and was managed with dual antiplatelet therapy consisting of 300 mg aspirin and 600 mg clopidogrel.

*Case 4. Internal Cervical Carotid Artery Pseudoaneurysm*** ** 

**Figure 4:**
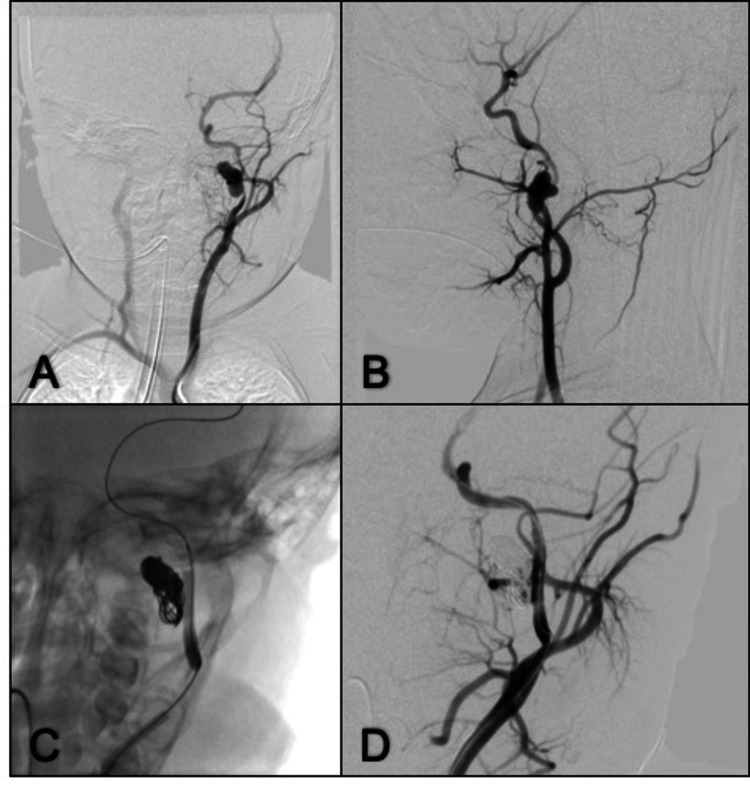
Anterior-posterior and lateral left CCA angiogram (A, B) A lobulated pseudoaneurysm is seen at proximal left ICA, vessel wall narrowing is noted; (C) Coils were deployed inside the sac, followed by stent placement at the level of the neck pseudoaneurysm; (D) Control left CCA angiogram depicts complete obliteration of the pseudoaneurysm. CCA: common carotid artery. ICA: internal carotid artery

A one-year-old girl presented to pediatrics with pain and swelling over the upper left side of the neck, accompanied by acute upper respiratory infection symptoms. She was initially diagnosed with a retropharyngeal abscess drained by puncture twice. Ten days later, she suddenly presented with intense oropharyngeal bleeding that caused hypovolemic shock. An angiography was performed, which revealed a pseudo aneurysm of the C1 segment of the left internal carotid artery (15 x 6.5 mm). It was embolized with two titanium coils (Axium™ 3D, Medtronic PLC; 14 mm x 40cm and 8 mm x 30 cm), then an angioplasty was performed with a self-expandable stent (Liberté Monorail, Boston Scientific Corporation; 3mm x 32mm). Angiographic projections reported obliteration of the pseudoaneurysm. In the postoperative period, the patient was discharged on the second postoperative day and followed up in the Endovascular Department. The one-year angiography follow-up showed no recurrence of the lesion (Figure 4).

Arteriovenous fistula

The arteriovenous fistula is defined as an abnormal communication between the high-flow arterial system and the low-flow venous network, which directly connects the fluent artery and the nearby draining veins without the normal intervention of the capillary bed [2].

Two mechanisms of formation of post-traumatic arteriovenous fistula are described: The theory of simultaneous artery lacerations and the accompanying vein that results in a single fistula [5]. The other is the theory of the disruption of the vasa vasorum of the arterial wall, which proposes the proliferation of endothelial cells from the vasa vasorum towards the surrounding hematoma; interrupted vasa vasorum form endothelial thickenings of numerous small vessels [6,7] resulting in multiple vascular channels created for adjacent veins. Angiography continues to be the study of choice for diagnosing these lesions, but magnetic resonance imaging and CT can provide hemodynamic data less invasively [8,9]. In addition, embolization has been proposed as a safer alternative to surgical ligation or resection in such situations [2].

*Case 5. High Flow Right Jugulo-Carotid Arteriovenous Fistula * 

**Figure 5:**
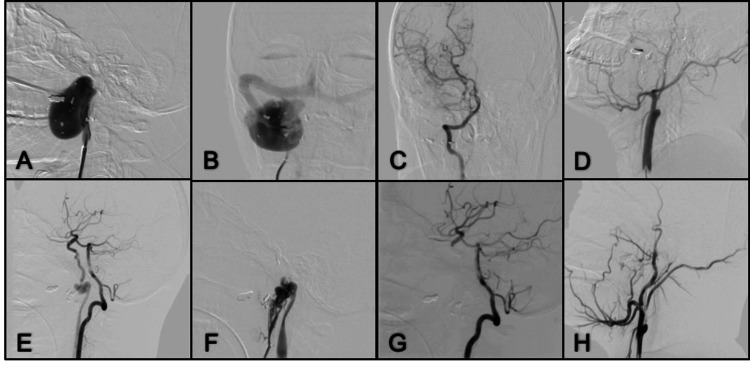
Lateral and anterior-posterior projection of DSA (A, B) Right ICA with abnormal communication towards the internal jugular vein and presence of pseudoaneurysm; (C) Anterior-posterior image with right VA angiogram showing the presence of a right PCom artery that provides intracranial flow to the terminal branches of the right ICA; (D) Right CCA angiogram after surgical closure of the right ICA without evidence of pseudoaneurysm; (E) Follow-up image with right VA angiogram in lateral view where recanalization of the fistula is observed through the PCom; (F) ECA angiogram shows communication to the jugular vein through the ascending pharynx, which was later embolized; (G) Control left VA angiogram in lateral view with occlusion of the cavernous segment of the right ICA; (H) CCA control angiogram in lateral projection with complete fistula closure to the right internal jugular vein. DSA: digital subtraction angiography; AP: anterior-posterior; VA: vertebral artery; PCom: posterior communicating; ECA: external carotid artery; CCA: common carotid artery; ICA: internal carotid artery

A 21-year-old male patient presented at the emergency room with a head gunshot injury in the right upper cervical region. Initial management included only surgical debridement and primary wound closure. Three months later, he presented with right eye chemosis and conjunctival hyperemia. CTA was performed showing pseudoaneurysm (51 x 40 mm) and high flow right jugular-carotid arteriovenous fistula. Surgical closure of the right internal carotid at the upper cervical segment was performed. Postoperative angiogram identified persisting filling of the fistula due to retrograde flow coming from the posterior communicating artery, at the cavernous part of the right internal carotid. A 5 x 15 coil was deployed. Nonadhesive liquid embolic (Onyx) was administered to obtain complete vessel occlusion. After seven months of follow-up, the patient remained asymptomatic, but the control angiogram identified fistula recurrence through filling from ascending pharyngeal artery and posterior communicating artery; the second session of embolization with Onyx 18 obtained 100% occlusion (Figure 5).

Case 6. Right Superficial Temporal Artery and the Ipsilateral Facial Vein Arteriovenous Fistula 

**Figure 6:**
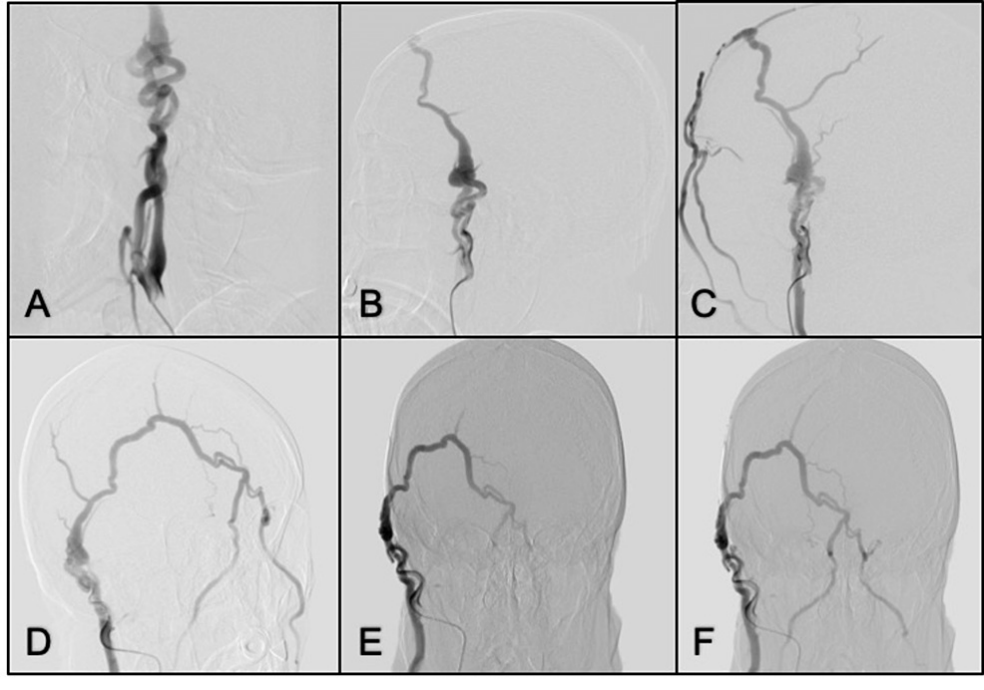
Lateral view CCA angiogram (A) Early arterial, (B) late arterial, and (C) venous phase, there is an abnormal communication between the right superficial temporal artery and the ipsilateral facial vein; there is no intracranial circulation involvement. (D) Oblique, (E) right, and (F) anterior-posterior in the venous phase show drainage to both facial veins. CCA: common carotid artery

A 71-year-old male patient presented with four months of local right preauricular increase in volume, with audible bruit, palpable pulsation, and thrill. Angiotomography and brain angiogram revealed an arteriovenous fistula between the right superficial temporal artery and the ipsilateral facial vein without any involvement of internal carotid circulation (Figure 6). Open surgery was scheduled for surgical fistula resection; histopathological analysis confirmed the diagnosis. Till the six-month follow-up, there was no clinical recurrence.

Case 7. Right Temporal Superficial Artery Arteriovenous Fistula 

**Figure 7:**
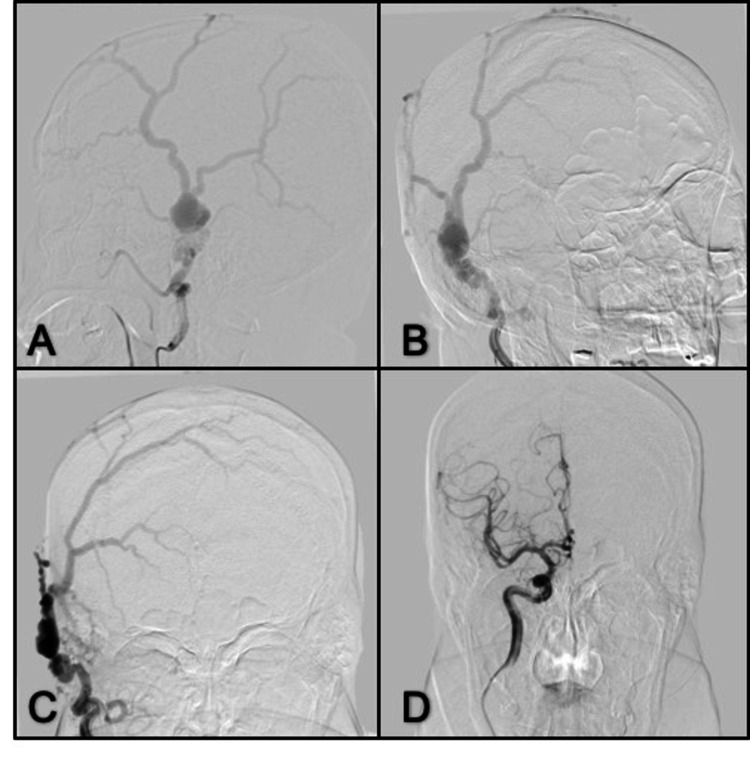
Lateral, oblique, right, and anterior-posterior views from right ECA angiogram (A, B, C) Dilated temporal superficial artery in late venous phase drainage to dilated veins; (D) Anterior-posterior view of the internal carotid artery angiogram shows no intracranial vessel involvement. ECA: external carotid artery

A 62-year-old male with no history of traumatic lesions developed a pulsatile lesion over the right temporal region of the scalp. When first seen in the clinic, a 3.5 x 2.5 mass in the temporal area was painless to palpation with slight thrill. This mass appeared gradually over six months. A diagnostic arteriography, which reports an arteriovenous fistula of the right temporal superficial artery, was performed (Figure 7). The complete surgical excision was performed three months later. The patient was sedated with general anesthesia, the region was infiltrated, and a Souttar incision was made. Meticulous dissection of the lesion was made, and the right temporal superficial artery was clipped at the level of the external carotid artery. Emissary frontal veins were coagulated, and the lesion was completely resected. The patient was discharged on the second postoperative day with slight palsy of the temporal branch of the right facial nerve and followed up in the Neurosurgery Department. The patient remained symptom-free at six months postoperatively.

Case 8. Left Temporal Superficial Artery Arteriovenous Fistula

**Figure 8:**
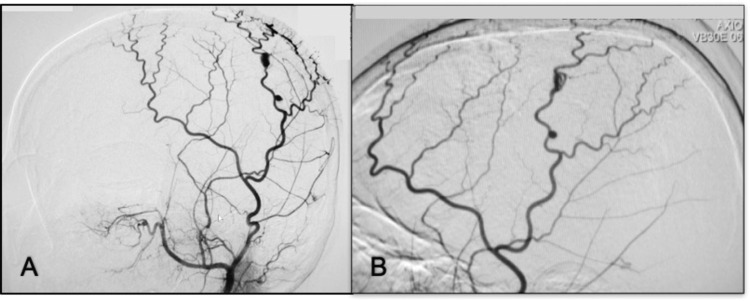
Lateral view left ECA angiogram (A, B) Fistula from the left superficial temporal artery ECA: external carotid artery

A 25-year-old man with a history of mild cranioencephalic trauma developed a pulsatile and tortuous lesion over the left parietal region of the scalp. When first seen in the hospital, there is a vascular lesion with several areas of dilatation with palpable thrill and pain to the palpation of the parietal region. The lesions had appeared and grown up gradually over three months. A diagnostic arteriography, which reports an arteriovenous fistula of the left temporal superficial artery, was performed (Figure 8). Surgical treatment was completed two months later, the artery was clipped, and the lesion was resected. The patient was discharged on the second postoperative day with no complications. He was lost to follow-up in our department.

Case 9. Temporal Superficial Artery Arteriovenous Fistula 

**Figure 9:**
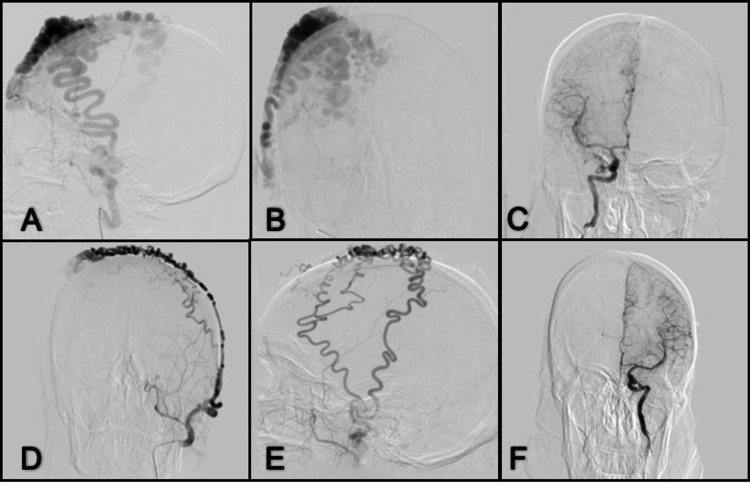
Lateral and anterior-posterior view of left ECA angiogram (A, B) Ectatic superficial temporal artery with abnormal venous drainage; (C) Left ICA angiogram without evidence of a connection to extracranial fistula; (D. E) Anterior-posterior and lateral view of right ECA angiogram show ectatic superficial temporal artery with abnormal venous drainage to the left side; (F) Right ICA angiogram absence of abnormal connection to abnormal extracranial vessels. ECA: external carotid artery; ICA: internal carotid artery

A 71-year-old woman with no history of traumatic lesions developed a bilateral pulsatile tortuous large mass over the frontal and parietal regions of the scalp. This mass had appeared and grown up slowly and gradually over three years. When seen in the clinic, there was a tortuous lesion on bilateral frontoparietal regions, with thrill to palpation and no pain. It deformed the scalp and forehead surface. A diagnostic arteriography was performed, which reported arteriovenous fistulae of both superficial temporal arteries (Figure 9). The complete surgical excision was performed two months later. Meticulous dissection of the vascular lesion was made and resected. The patient was discharged on the fourth postoperative day with no neurological impairment and followed up in the Neurosurgery Department. The patient remained symptom-free for three years postoperatively.

Dural fistulas

Intracranial dural AVFs represent 10-15% of all intracranial vascular malformations. Although dural AVFs can occur anywhere in the dura mater covering the brain, they occur most frequently in the cavernous, transverse, and sigmoid sinuses. Patients may be asymptomatic or experience symptoms ranging from mild to fatal hemorrhage. Furthermore, these symptoms may be characterized as either nonaggressive (benign) (e.g., tinnitus) or aggressive (e.g., intracranial hemorrhage, neurologic deficits). Although several classification systems have been developed to grade the risks of dural AVFs, those devised by Cognard et al. and Borden et al. are the most widely used [4,10],.

Case 10. Right Temporo-Occipital Dural Fistula 

**Figure 10:**
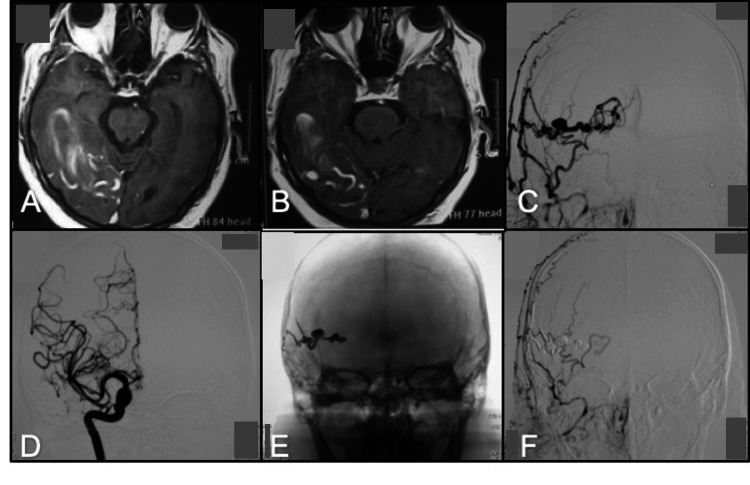
MRI axial T1 (A, B) Abnormal dilated vessels and hemorrhage; (C ) Anterior-posterior view, right ECA angiogram shows feeding arteries from the superficial temporal artery and occipital artery; (D) Right ICA angiogram shows no feeding arteries; (E) Fluoroscopic image depicting the final cast distribution of Onyx-18; (F) Right external carotid artery angiogram after embolization of the fistula with complete occlusion. ECA: external carotid artery; ICA; internal carotid artery

A 56-year-old male with no personal pathological history, and no history of trauma, attended an evaluation due to a headache with a long evolution in the right hemicrania, pulsatile, which increased with the Valsalva maneuver and one year before an increase in volume in the right temporal region and dizziness accompanied admission. Diagnostic angiography was performed, finding a right temporo-occipital dural fistula whose pedicles came from distal branches of the right temporal superficial artery, posterior auricular, and occipital artery with retrograde reflux from the posterior third of the superior sagittal sinus. The entire lesion was embolized with Onyx (Figure 10). He was discharged on the second day of the procedure without complications.

Case 11. Post Surgical Left Temporoparieto-Occipital Dural Fistula 

**Figure 11:**
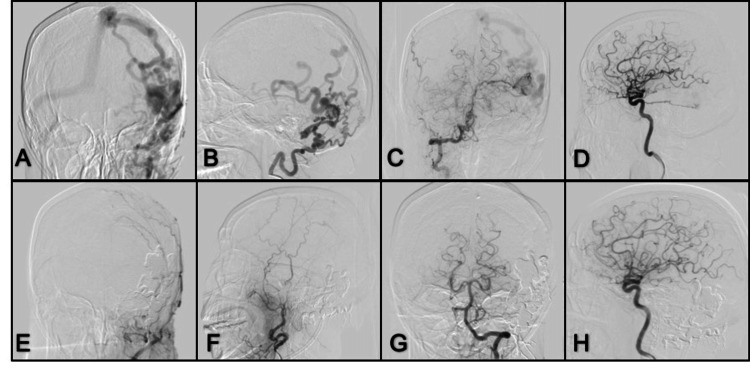
Anterior-posterior and lateral view right ECA angiogram (A, B) Dural occipital fistula with feeding arteries from occipital artery and abnormal venous drainage to dilated cortical veins and superior sagittal sinus, (C) right VA angiogram shows feeding arteries from the PCA; (D) Left ICA angiogram shows feeding arteries from the meningohypophyseal trunk; (E, F) Right ECA angiogram after embolization anterior-posterior and lateral view after complete occlusion of the dural fistula using Onyx-18; (G, H) Control angiogram from the right VA and left ICA. VA: vertebral artery; PCA: posterior cerebral artery; ICA: internal carotid artery; ECA: external carotid artery

A 46-year-old female with no significant history who reported symptoms seven years ago with severe, progressive, disabling headache, was treated with analgesics without improvement. A simple skull tomography was performed and contrasted by symptoms, finding an image compatible with arteriovenous malformation (AVM), which is why a resection was performed without complications. Later, one year ago, she again presented an oppressive headache, predominantly biparietal holocranial, accompanied by blurred vision, increased with supine decubitus without mitigating, with slight improvement after the administration of non-steroidal analgesics. Angiography was performed where a left dural temporoparieto-occipital fistula was documented through the ipsilateral meningeal-pituitary plexus (Figure 11). Onyx embolization was performed without complications. The patient was discharged on the third day.

Case 12. Carotid-Cavernous Fistula (Barrow type D) 

**Figure 12:**
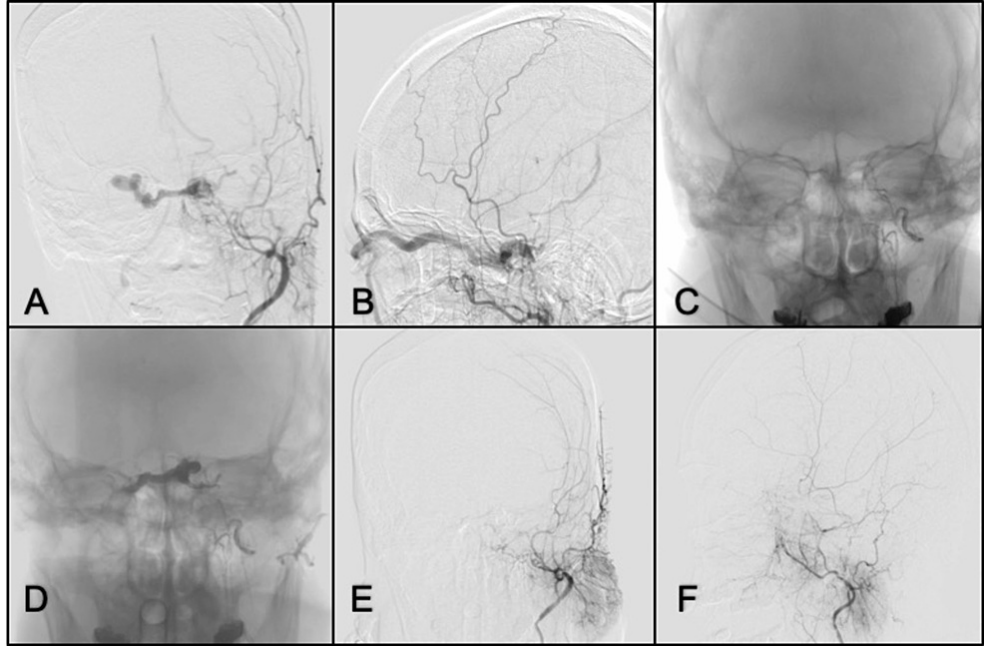
Anterior-posterior and lateral views of left ECA angiogram (A, B) Abnormal drainage to the cavernous sinus and the ophthalmic vein; (C, D) Fluoroscopic image depicting the cast distribution of Onyx-18; (E, F) Anterior-posterior and lateral views of the left ECA angiogram after embolization of the fistula show complete occlusion. ECA: external carotid artery

A 52-year-old female presented with progressive blurred vision, proptosis, and chemosis of the left eye, with a history of mild cranioencephalic high-energy trauma with no intracranial injury one month before the onset of symptoms. She consulted in the Neurosurgery Department two years before the trauma. Angiography showed a carotid-cavernous fistula (Barrow type D). The fistula was embolized with Onyx from the left external carotid artery (Figure 12). The patient was discharged on the second post-angiography day with no neurological impairment and followed up in the Neurosurgery Department. The patient remained symptom-free for two years post embolization.

Case 13. Left Dural Arteriovenous Fistula

**Figure 13:**
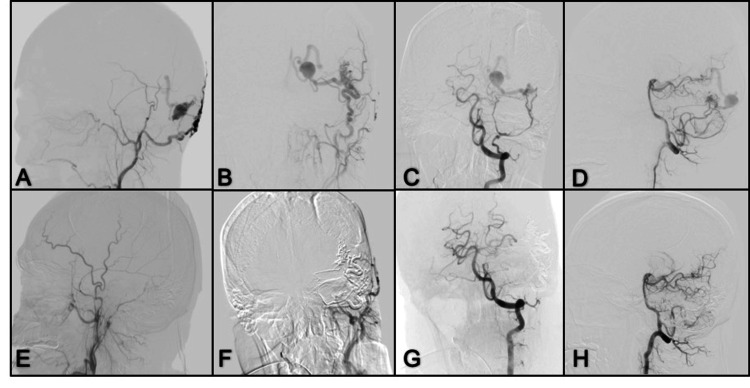
ECA angiogram (A, B) Lateral and anterior-poterior view left ECA angiogram; (C, D) Anterior-posterior and lateral view of left VA show dural occipital fistula with feeding arteries from occipital artery and PICA; (E, F) Lateral and anterior-posterior view left ECA control angiogram after Onyx embolization through the occipital artery; (G, H) Anterior-posterior and lateral left VA angiogram with complete fistula occlusion. PICA: posterior inferior cerebellar artery; ECA: external carotid artery; VA: vertebral artery

A 48-year-old male with a history of hypertension with adequate control went to the emergency department due to a long-term, holocranial, throbbing headache, which had increased in intensity in the last six months and sometimes woke him up from sleep. It was accompanied by increased volume in the occipital region. After administering analgesics, the patient presented to the emergency room again for increased pain without improvement. Angiography was performed, finding left dural arteriovenous fistula Cognard grade 4 dependent on external carotid, occipital, and left vertebral branch, which was embolized with nonadhesive liquid embolic (Onyx) without complications, and the patient was discharged on the second day after the procedure (Figure 13).

Pial fistulas

Pial intracranial arteriovenous fistulas, which do not involve the vein of Galen, are a direct connection between an artery and a vein without a vascular nest. The characteristic presentation is a single feeding artery with an aneurysmal venous dilatation. These types of fistulas are rare, and their understanding is limited. Therefore, at the usual age of presentation in pediatric patients or young adults, the clinical history should be interrogated in the search for a history of trauma. They require aggressive treatment; it is not common for them to present spontaneous regression, and the prognosis with conservative management is poor. Current treatments include surgical resection and endovascular embolization.

Case 14. Medial Cerebral Artery Pial Fistula 

**Figure 14:**
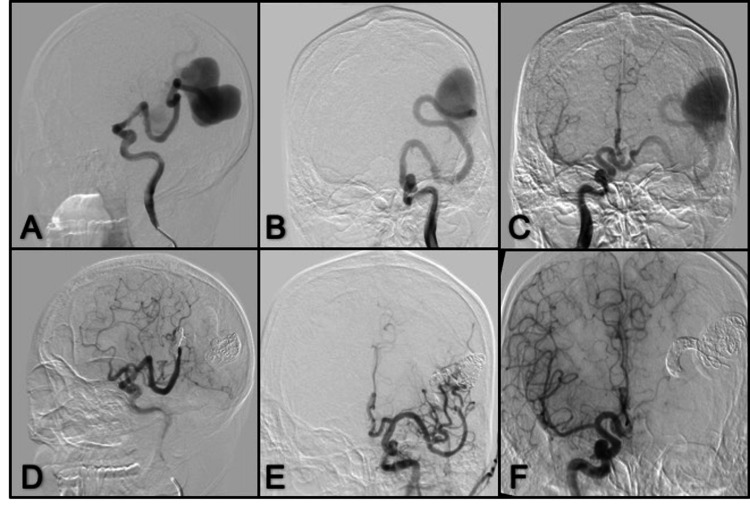
DSA lateral and anterior-posterior projection of the left ICA angiogram in early arterial phase. (A, B) Presence of direct communication with venous ectasia; (C) Anterior-posterior right ICA angiogram shows recruitment even from the contralateral side and poor opacification of cortical branches; (D, E) Anterior-posterior and lateral projection in the late arterial phase of left ICA angiogram after embolization with liquid embolic and coils; there is opacification of ACA and branches from MCA; (F) Anterior-posterior control angiography of the right ICA with a good filling of cortical branches on the left side. ICA: internal carotid artery; ACA: anterior cerebral artery; MCA: medial cerebral artery; DSA: digital subtraction angiography

An 18-year-old male had a history of generalized tonic-clonic seizures six times in the past four years before he has first seen in the clinic and treated with phenytoin and valproic acid. MRI reported left front-parietal-occipital vascular lesions. He did not have any neurological impairment. Angiography showed a pial fistula connecting the medial cerebral artery and superior sagittal sinus with giant venous ectasia (Figure 14). It was embolized with one coil and liquid embolic (Onyx). The patient was discharged on the fourth postoperative day and followed up in the Endovascular Department.

*Case 15. Right Pericallous Artery Pial Fistula* 

**Figure 15:**
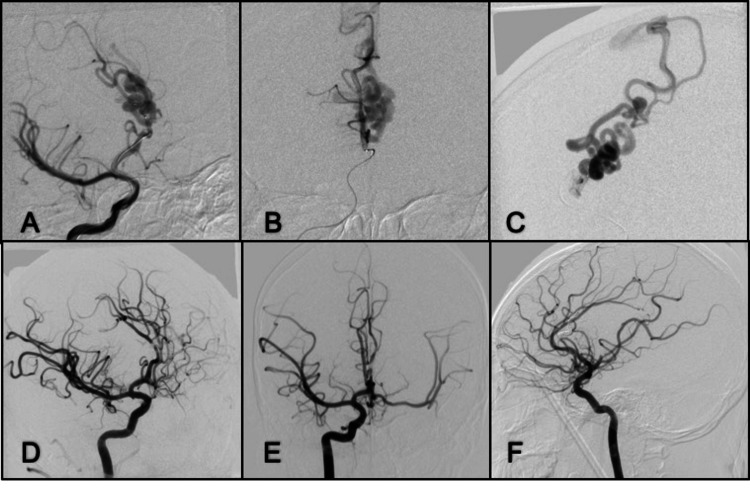
Oblique view of right ICA angiogram early arterial phase. (A) A direct communication from right ACA to ectatic cortical veins; (B, C) Anterior-posterior and lateral view right ACA microcatheter angiogram, drainage to the SSS; (D, E, F) Oblique, anterior-posterior, and lateral views right ICA control angiograms with complete obliteration of the fistula using liquid embolic (Onyx-18). ICA: internal carotid artery; ACA: anterior cerebral artery; SSS: superior sagittal sinus

A 10-year-old female patient presented at the emergency department with seizures, intense headache, and 10/15-point Glasgow coma scale. CT showed intraparenchymal hemorrhage in the right frontal lobe, with intraventricular extension that required management with external ventricular drain for seven days. After clinical conditions improved, the ventricular drain was retired. A brain angiogram identified a right fistulous connection between the right perilous artery and superior sagittal sinus, diagnosed as pial fistula. The large ectatic venous dilatation or venous varices may be in the form of multiple pouches resembling a nidus with intranidal aneurysms, as shown in this case. The fistulous malformation was embolized using a nonadhesive liquid embolic (Onyx 18) (Figure 15). One year later, control angiography revealed no recurrence or residual lesion, and the patient presented full recovery.

## Discussion

This series of selected cases highlights unique vascular lesions with a late presentation, some with a traumatic background, and sometimes even iatrogenic.

In the case of pseudoaneurysm, angiography should be performed as soon as possible after encountering a high-risk patient [11]. CTA is helpful for evaluating these injuries, but it cannot necessarily be considered a definitive evaluation; the diagnosis must be tailored to individual circumstances. CTA was only 80% sensitive in detecting pseudoaneurysms [11,12]. A CTA is recommended in all patients with skull base fracture and intracranial hemorrhage [8], with pseudoaneurysms being the most frequent finding in the late evaluation and can result in life-threatening episodes of rebleeding [12]. These can cause intracranial hemorrhages in up to 60% of affected patients, and the mortality rate is 31-54% [13]. Endovascular techniques, in most cases, offer the most significant simple and low-risk treatment option. The endoluminal approach is attractive since cannulation of even distal external carotid artery branches in young, healthy patients with non-atherosclerotic trauma is usually straightforward [12].

Direct surgical repair of these lesions is not often possible, involving extensive dissection with the risk of injuring adjacent structures. Although the use of covered stent for pseudoaneurysm has been previously reported, here we show the combined use with coils and Onyx with two objectives: first reducing the amount of material needed for complete occlusion of the lesion by focusing on the narrowest part of the anomalous communication and as a second "rescue option" when endoleak is present.

It is important to identify pial fistulas by remembering that a single afferent artery is usually involved, sometimes several, and a varicose dilation or a venous aneurysm. There is no involvement of dural vessels. It can be challenging to differentiate from an AVM when there is more than one feeding artery; venous ectasia can simulate a "false nest" like AVMs and pial fistulas are located in the parenchyma. The ideal treatment is only the closure of the artery-vein shunt, which can be achieved only using EVOH or with the fusion of coils + EVOH.

## Conclusions

A broad spectrum of lesions can be presented as late trauma complications; endovascular treatment is a safe and effective approach for both intracranial and extracranial lesions. Endovascular treatment can be a single option or adjuvant to other hybrid therapies. We have described standard techniques to treat different vascular pathologies, sometimes with limited resources. Although the development of these lesions is not usual,it is important to be aware of the option of endovascular treatment sometimes as the initial approach.
